# Supplementary material for: Cloud BioLinux: pre-configured and on-demand bioinformatics computing for the genomics community
Source: BMC Bioinformatics. 2012 Mar 19;13:42. doi: 10.1186/1471-2105-13-42 (PMC3372431; doi:10.1186/1471-2105-13-42)
Supplement: Additional file 1 — Supplementary 1 Cloud BioLinux software documentation in the form of a mini, self-contained website. Users need to download and uncompress the .zip file, and open through a web browser the "index.html" file available on the main directory. (ZIP 1823 kb). [file 1471-2105-13-42-S1.ZIP › Cloud-BioLinux-Package-Documentation/docs/dendroscope.html]

Bio-Linux Software Documentation Pages

Back to search form

## dendroscope

|  |  |
| --- | --- |
| Name | dendroscope |
| Description | Dendroscope is an interactive viewer for large phylogenetic trees. Researchers studying phylogenetic relationships need software that is able to visualize trees efficiently, increasingly of large datasets involving hundreds of thousands of taxa. The program should be user friendly (easy to run on all popular operating systems), facilitate interactive browsing and editing the trees and allow one to export the result in multiple file formats in publication quality. |
| Homepage | http://www-ab.informatik.uni-tuebingen.de/software/dendroscope/welcome.html |
| Remote Documentation |  |
